# A Pilot Analysis of Whole Transcriptome of Human Cryopreserved Sperm

**DOI:** 10.3390/ijms25074131

**Published:** 2024-04-08

**Authors:** Sara Stigliani, Adriana Amaro, Francesco Reggiani, Elena Maccarini, Claudia Massarotti, Matteo Lambertini, Paola Anserini, Paola Scaruffi

**Affiliations:** 1 SS Physiopathology of Human Reproduction, IRCCS Ospedale Policlinico San Martino, Largo R. Benzi 10, 16132 Genova, Italy; sara.stigliani@hsanmartino.it (S.S.); claudia.massarotti@unige.it (C.M.); paola.anserini@hsanmartino.it (P.A.); 2 SSD Regolazione dell’Espressione Genica, IRCCS Ospedale Policlinico San Martino, 16132 Genova, Italy; 3 Department of Neuroscience, Rehabilitation, Ophthalmology, Genetics and Maternal-Child Health (DiNOGMI), University of Genova, 16132 Genova, Italy; 4 Department of Internal Medicine and Medical Specialties (DiMI), University of Genova, 16132 Genova, Italy; matteo.lambertini@unige.it; 5 Department of Medical Oncology, UOC Clinica di Oncologia Medica, IRCCS Ospedale Policlinico San Martino, 16132 Genova, Italy

**Keywords:** sperm, sperm cryopreservation, human sperm, fertility preservation, microarray, transcriptome, sperm donation

## Abstract

Sperm cryopreservation is a procedure widely used to store gametes for later use, to preserve fertility in patients prior to gonadotoxic treatments or surgery, and for sperm donation programs. The purpose of the study was to assess the impact of cryopreservation on human sperm transcriptome. Semen samples were collected from 13 normospermic men. Each sample was divided into two aliquots. The total RNA was immediately extracted from one aliquot. The second aliquot was frozen and total RNA was extracted after a week of storage in liquid nitrogen. The RNA samples were randomized in four pools, each of six donors, and analyzed by microarrays. The paired Significance Analysis of Microarray was performed. We found 219 lower abundant transcripts and 28 higher abundant transcripts in cryopreserved sperm than fresh sperm. The gene ontology analysis disclosed that cryopreservation alters transcripts of pathways important for fertility (i.e., spermatogenesis, sperm motility, mitochondria function, fertilization, calcium homeostasis, cell differentiation, and early embryo development), although the increase of some transcripts involved in immune response can compensate for the harmful effects of freezing.

## 1. Introduction

Cryopreservation of human spermatozoa in liquid nitrogen is routinely used in the assisted reproduction technology (ART) laboratory for reasons that include ensuring the availability of sperm on the day of insemination or in vitro fertilization in case of unavailability of the male partner or problems in semen collection due to stress factors and anxiety; fertility preservation in patients who develop gonadal failure and men at risk of losing fertility due to gonadotoxic therapies or surgery; and for sperm donation programs.

The two main sperm cryopreservation methods currently available are conventional freezing (namely, slow and rapid freezing) and vitrification [1]. In slow freezing, spermatozoa are mixed with cryoprotectants and cooled progressively for 2–4 h in two or three steps, manually or automatically, using a programmable machine. In the rapid freezing technique, the sperm–cryoprotectant suspension loaded into a cryo-straw is exposed to a liquid nitrogen vapor phase for about 10 min before being plunged into liquid nitrogen [2]. Vitrification is an alternative method in which the sperm suspension is plunged directly into liquid nitrogen and the sperms are cooled ultra-quickly [3].

Human spermatozoa tolerate a range of cooling and warming rates, due to the high fluidity of the membrane from the unsaturated fatty acids in the lipid bilayer and their low water content [4]. Cell survival after freezing and thawing largely depends on minimizing the formation of intracellular ice crystals. This is done by using appropriate cryoprotectants and applying cooling and warming rates that minimize the amount of intracellular water subject to ice formation. Several cryoprotectants are commercially available and are classified as permeating (such as dimethyl sulfoxide and glycerol) and nonpermeating (such as albumins, dextrans, and egg yolk citrate) [5].

Despite its widespread use, the cryopreservation technique can cause damage to sperm through oxidative stress that induces lipid peroxidation, DNA fragmentation, or apoptosis [6]. Moreover, sperm motility and fertilizing potentials are reduced due to membrane, cytoskeletal, and acrosome damages [7,8]. Transcript changes have been reported in cryopreserved sperm from mice [9], boars [10,11], bulls [12,13], and giant pandas [14]. It has been reported that cryopreserved human sperm shows a decrease in levels of *CATSPER2* and *TEKT2*, which are involved in sperm motility [15], and of *SPAG5*, *SPAG7*, and *SPAG12* related to the positive outcome of ART [16]. Valcarce et al. [17] demonstrated that transcripts considered human spermatozoa quality markers (*PRM1*, *PRM2*, and *PEG1*/*MEST*) and markers for pregnancy success (*ADD1*) were reduced after conventional cryopreservation. In a comparison of transcript profiles in fresh, frozen, and vitrified human spermatozoa, Wang et al. found that the conventional freezing process induces more transcript alterations than vitrification and most of these pathways are relevant for apoptosis and immune response [18]. Very recently, a multiomics analysis confirmed that conventional cryopreservation significantly reduced sperm motility and mitochondrial structure [19].

Although the vitrification of spermatozoa is emerging as safer than the conventional slow freezing, it should currently be considered an experimental procedure [1] and slow freezing is the routine cryopreservation used in clinics. All over the world, there is a huge amount of semen samples cryopreserved after slow cooling, and laboratories keep freezing like this. To date, studies published on the transcriptomic profile of fresh and conventional frozen human sperm have not recruited a large number of donors (4 by Wang et al. [18] and 15 by Fu et al. [19]). Considering these factors, we believe there is still room to analyze other normozoospermic donors using high-throughput technology. This may confirm previously reported results and/or identify other transcript changes associated with conventional cryopreservation.

For this purpose, we compared the wide transcript profiles of 13 paired fresh and cryopreserved sperm samples using a microarray approach.

## 2. Results

**Figure 1:**
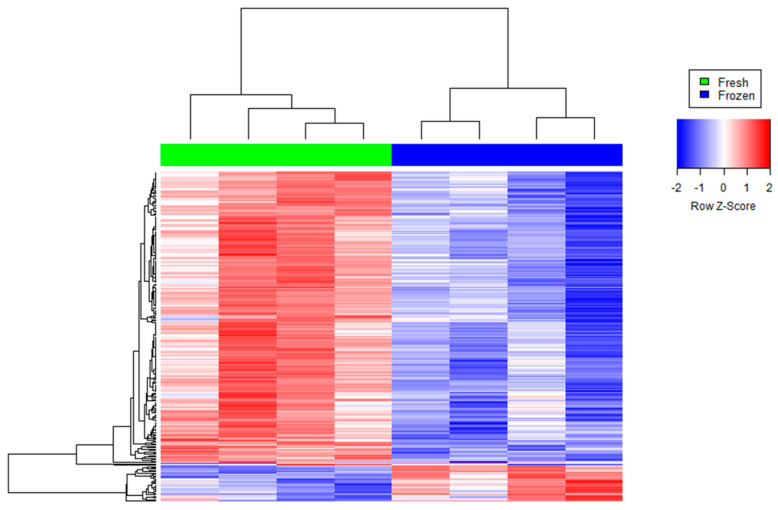
Agglomerative hierarchical clustered heat map of differentially abundant transcripts in cryopreserved (“frozen” samples in blue) versus non-cryopreserved (“fresh” samples in green) sperm. Each color patch represents the amount of transcripts (row) in that sample (column), with a continuum of levels from bright blue (lowest) to bright red (highest).

**Figure 2:**
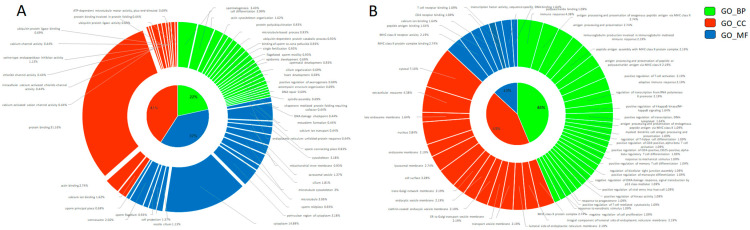
Gene Ontology donut plot of differentially abundant transcripts between fresh and cryopreserved spermatozoa. All three components (MF = molecular function, BP = biological process, and CC = cellular components) are represented in the donut chart in blue, orange, and green, respectively. (**A**) Decreased transcripts. (**B**) Increased transcripts. The donut charts were obtained with Ggplot2 package of R software (v. 4.2.1).

We found 219 reduced transcripts and 28 increased transcripts in cryopreserved sperm than fresh sperm (Figure 1, Tables S1 and S2). The altered transcripts in cryopreserved sperm were annotated for GO terms to identify their potential role in molecular function (MF), biological process (BP), and cellular components (CC). The most significantly (*p* ≤ 0.05) enriched BP, MF, and CC pathways were selected based on the counts of genes involved in each GO category (n ≥ 3) and the type of analyzed cells (spermatozoa) (Figure 2). By combining all these data, the reduced transcripts after cryopreservation were mainly involved in spermatogenesis (*CABS1* (Calcium Binding Protein, Spermatid Associated 1), *CCIN*, *HERPUD2*, *LIMK2*, *MEA1*, *OAZ3*, *ODF2*, *PROK2*, *SPATA6*, *SPATA6L*, *SPATA19*, *SPATA32*, *SPMIP6*, *TBC1D21*, *TXNDC2*); in sperm motility by the correct organization of cytoskeleton in the sperm midpiece and tail (*ACTRT1*, *ACTRT2*, *AKAP4*, *AP1*, *CABS1*, *CAPZA3*, *CAPZB*, *CCIN*, *DNAL4*, *DYNLL2*, *DYRK4*, *EPB41L2*, *FAM161B*, *IFT172*, *IQCG*, *KIF2B*, *LIMK2*, *LYST*, *MAPRE3*, *NF2*, *ODF2*, *PHACTR1*, *SAXO1*, *SGCA*, *SPATA6*, *SPATA6L*, *SPATA19*, *TBC1D21*, *TEX35*, *TUBA4A*, *TUBA8*) and of the mitochondria function (*CHCHD3*, *SPATA19*, *TBC1D21*); in fertilization by deregulating the oocyte activation (*PLCZ1*), the acrosome vesical (*ACTL7A*, *CABS1*, *IQCF1*, *PRSS37*, *TBC1D21*, *TUBA8*), the binding of sperm to zona pellucida (*HSPA1L*, *PRKAR2A*, *PRSS37*), and the spindle assembly (*MAPRE3*, *NCOR1*, *TUBGCP3)*; in calcium ion binding and homeostasis (*ALOX15B*, *AMY1C*, *ANKEF1*, *ANO1*, *ANO2*, *CABS1*, *CPNE9*, *ITPR3*, *KCNIP2*, *PLCZ1*, *PKD2L1*, *S100A4*, *SELENOK*); in cell differentiation (*CCIN*, *CPNE9*, *GLRX2*, *HEMGN*, *KRTDAP*, *MEA1*, *ODF2*, *SYAP1*, *SPATA6*, *SPATA19*, *SPMIP6*, *TCF4*, *TXNDC2*) and early embryo development (*CNN1*, *EPB41L2*, *GPI*, *IFT172*, *KRTDAP*, *MICAL2*, *NF2*, *NRDC*, *PHACTR1*, *POPDC3*, *ROBO1*, *SPRR2D*, *TIAM2*, *TNNI3*); protein polyubiquitination and catabolic process (*BAG1*, *HERPUD2*, *HSPA1L*, *MARCHF8*, *PSMA6*, *RNF133*, *STX8*, *TRIP12*, *UBE2DNL*, *UBL3*, *UBQLN3*); and chaperone-mediated protein folding and endoplasmic reticulum unfolded protein response (*BAG1*, *DNAJB8*, *HERPUD2*, *HSPA1L*, *SERP2*) (Table S3). The more important reduced transcripts in the cryopreserved spermatozoa were encoded by genes mostly localized in the cytoplasm and cytoskeleton at the level of the sperm flagellum (*AKAP4*, *CABS1*, *IFT172*, *IQCG*, *ODF2*, *SAXO1*, *SPATA19*, *TBC1D21*), acrosomal vesicle (*ACTL7A*, *CABS1*, *PRSS37*, *PLCZ1, TBC1D21*, *TUBA8*), and centrosome (*CEP170*, *CEP295NL*, *DYNLL2*, *ENTR1*, *KIF2B*, *KIZ*, *LIMK2*, *ODF2*, *PRKAR2A*, *TUBGCP3*, *TTC39A*).

The increased transcripts found in cryopreserved sperm were enriched in pathways related to immune response (*CD74*, *HLA-DMA*, *HLA-DRB1*, *HLA-DQB1*, *HLA-DRA*, *RGS1*, *THBS1)* and antigen processing and presentation via MHC class II (*CD74*, *HLA-DMA*, *HLA-DRB1*, *HLA-DQB1*, *HLA-DRA*) (Table S4). Consistently with their functions, the abundant transcripts in cryopreserved sperm were mostly localized in the MHC class II protein complex (*CD74*, *HLA-DMA*, *HLA-DRA*, *HLA-DRB1*, *HLA-DQB1*), transport vesicle membrane (*CD74*, *HLA-DRA*, *HLA-DRB1*, *HLA-DQB1*), cell surface (*CD74*, *HLA-DMA*, *HLA-DRA*, *THBS1*, *HLA-DRB1*, *HLA-DQB1*), lysosomal membrane (*CD74*, *HLA-DMA*, *HLA-DRA*, *HLA-DRB1*, *HLA-DQB1*), and extracellular exosome (*CD74*, *HLA-DMA*, *HLA-DRA*, *HLA-DRB1*, *HLA-DQB1*).

**Figure 3:**
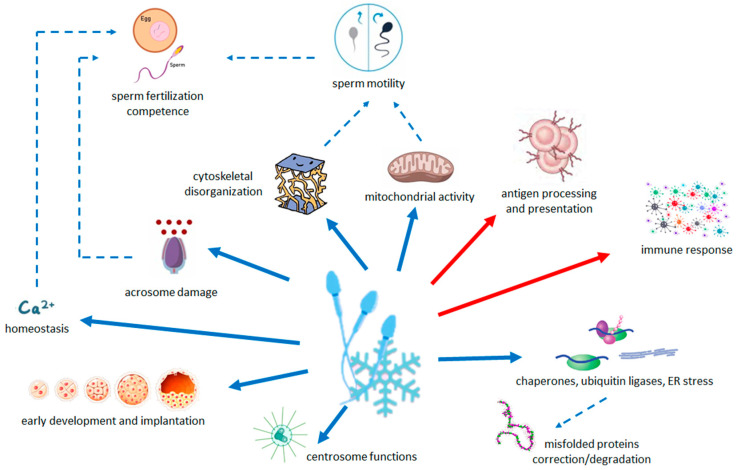
Schematic summary of the relationship between the pathways modified by cryopreservation and the quality and functions of sperm. Arrows with solid line indicate the less (blue) and more (red) abundant pathways in cryopreserved sperm with respect to fresh sperm. Dashed arrows indicate the processes that are decreased (blue) by changes in transcriptome.

Results are summarized in Figure 3.

## 3. Discussion

Cryopreservation alters sperm structure and function and causes DNA fragmentation and damage [20]. In this study we showed that cryopreservation induces changes in the abundance of the human sperm transcriptome. The number of decreased transcripts in cryopreserved spermatozoa was higher than those increased compared to fresh samples. These results agree with previous studies that identified a lower abundance of transcripts in frozen and vitrified sperm than fresh samples [18,19].

The most decreased transcripts after cryopreservation were linked to the organization of the cytoskeleton. Actin- and microtubule-modulating activities are involved in this context as well as sperm-specific processes for appropriate head morphology (*CCIN* gene) [21], assembly of the connecting piece (*SPATA6*) [22], and the main parts of the sperm tail (*ODF2*) [23]. Previous studies are consistent with our results. In particular, loss of ODF member 2 [24] and ODF3 [18] were decreased after slow freezing. Functionally, sperm motility may be impaired by the loss of scaffold proteins as components of the centrosome matrix which is necessary for maintaining the elastic structures and recoil of the sperm tail. Structural damage to the sperm cytoskeleton was found to be induced by cryopreservation in both low and normal quality semen samples, where distribution of alpha tubulin was restricted to the end of flagellum due to the weakness of tubulin-containing structures [25,26].

In the midpiece of mature mammalian spermatozoon, the flagellum mitochondria are abundant and produce the energy required for sperm motility [27]. We found that cryopreservation reduces transcripts of *CHCHD3* and *SPATA19*, two key genes involved in the mitochondria organization and function. Specifically, CHCHD3 is a component of MICOS, a large protein complex of the mitochondrial membrane that plays a crucial role in maintaining cristae junctions, inner membrane architecture, and the formation of contact sites with the outer membrane [28]. SPATA19 is involved in the organization of mitochondria. In its absence, male mice are infertile and their spermatic mitochondria do not function properly [29]. Mitochondrial dysfunctions in sperm of different species caused by cryopreservation have been reported [6]. Also, the recent multiomics analysis performed by Fu et al. [19] found that the mitochondria in cryopreserved sperm had an abnormal ultrastructure, which indicated mitochondrial dysfunction. The presence of structural and functional alterations in mitochondria from asthenozoospermic subjects confirms the important role played by these organelles in the energetic maintenance of sperm motility [30,31]. As a result, the mitochondrial deficits due to sperm cryopreservation may contribute to male fertility reduction by limiting sperm mobility.

Moreover, our data demonstrated that cryopreservation may directly reduce sperm fertilization competence. On the tip of the head of the mature spermatozoon is the acrosome which is a membrane-bound organelle of Golgi apparatus origin. It contains hydrolases, including hyaluronidase and acrosin [32]. The acrosomal reaction, an exocytic event induced by an influx of calcium, plays an essential role during fertilization, enabling spermatozoa to penetrate the zona pellucida and fuse with the plasma membrane of the oocyte [33]. Acrosomal defects have been reported to cause male infertility in humans [34]. As previously reported in ultrastructural studies [35], cryopreservation may cause defective acrosome shape and function through the downregulation of genes involved in acrosome formation and reaction (i.e., *ACTL7A*, *IQCF1*, *PLCZ1*, *CAPZA3*, and *CAPZB* genes). Among the transcripts that we found decreased in cryopreserved sperm, *TBC1D21*, *TUBA8*, and *ACTL7A* encode proteins involved in acrosome assembly. TBC1D21 may be involved in acrosome formation and cytoskeletal reorganization during spermiogenesis, possibly by regulating RAB3A activity [36]. ACTL7A, an actin-like protein, plays an important role in acrosomal attachment and ultrastructural integrity [37] as it may be involved in the adhesion of the acrosomal outer membrane to the nuclear surface that is mediated by the subacrosomic perinuclear theca, a structural scaffold for the sperm nucleus [38]. In addition, sperm of ACTL7A mutated mice and men showed reduced expression and abnormal localization of PLCZ1 as a potential cause of fertilization failure even after ICSI (Intracytoplasmic Sperm Injection) [39]. PLCZ1 is located in the acrosome and is responsible for generating calcium oscillations that induce oocyte activation and early embryonic development [40]. Although ICSI is the recommended treatment for most couples with cryopreserved sperm and ICSI generally allows fertilization rates from 70% to 80%, total fertilization failure (TFF), where all mature oocytes fail to fertilize, occurs in 1–3% of all ICSI cycles [41]. Although it is a very low rate, this failure is worthy of study since it leaves patients who have only frozen semen with few options to achieve genetically related offspring. The main cause of TFF after ICSI is oocyte activation deficiency (OAD). In recent years, OAD has been associated with numerous PLCZ1 abnormalities, including gene mutations, reduced expression levels, or abnormal localization [42]. Cryopreserved sperm can have a negative regulation of acrosomal reaction and sperm motility through the reduction of *IQCF1*, which encodes a protein located in the acrosomal vesicle [43]. Further, the *CAPZA3* and *CAPZB* decreased in frozen–warmed spermatozoa are members of the F-actin capping protein (CAPZ) family, which assembles and disassembles the outer acrosomal membrane during capacitation. Similarly to our study, Wang et al. [18] found that CAPZA3 involved in the spermatozoa–oocyte interaction was decreased after slow freezing.

Calcium acts as an intracellular second messenger and its optimal seminal concentration is necessary for many physiological processes, including spermatogenesis, sperm motility, capacitation, acrosomal reaction, and fertilization [44]. Cryoinjury modifies membrane permeability to certain ions such as calcium and several studies have reported changes in intracellular calcium concentration in frozen–thawed sperm compared to fresh sperm [45,46,47]. The intracellular calcium is increased immediately after thawing and after a few minutes it decreases due to its extrusion from the disintegrated cell membranes induced by the cryopreservation–thawing process [48]. Based on these findings, changes in calcium ion binding and homeostasis may be another mechanism by which cryopreservation can weaken sperm function and all the steps leading to successful fertilization. We found that calcium-signaling-related processes can be deregulated in cryopreserved sperms by reduced levels of transcripts involved in calcium-regulated channels (*ANO1*, *ANO2*, *KCNIP2*, *ITPR3*), calcium oscillations (*PLCZ1*, *SELENOK)*, and calcium binding (*S100A4*, *ALOX15B*, *AMY1C*, *ANKEF1*, *CABS1*, *CPNE9*, *KCNIP2*).

Another analogy with previous transcriptome studies after sperm cryopreservation was the reduction of transcript sets of the endoplasmic reticulum-associated protein degradation process. In particular, we found the reduction of *HSPA1L* and *DNAJB8* (*HSP40*) transcripts as well as Wang et al. [18] observed a down-regulation of HSP90AA1, HSPA5, HSPA6, DNAJC3, DNAJC5B, and DNAJB11 after slow freezing. This HSP susceptibility to cryo-damage may correlate with the occurrence of apoptosis or necrosis due to improper handling of misfolded proteins. Decreases in HSPs preceded decreased motility in cooled and frozen spermatozoa [49,50]. HSP40, like HSP90AA1 in Wang et al.’s study [18], also has an ATPase activity. Therefore, it is observed that the common decreased motility of post-thaw spermatozoa may also be due to decreased HSPs, leading to limited ATP availability [50].

As a novelty of this study compared to previous omics analyses of cryopreserved human spermatozoa [18,19], we identified a reduction in transcripts involved in early embryo development, known to be affected by DNA damage after human sperm cryopreservation [20]. In particular, we found low levels of several transcripts involved in cell differentiation (*CCIN*, *CPNE9*, *GLRX2*, *HEMGN*, *KRTDAP*, *MEA1*, *ODF2*, *SYAP1*, *SPATA6*, *SPATA19*, *SPMIP6*, *TCF4*, *TXNDC2*) and early embryo development (*CNN1*, *EPB41L2*, *GPI*, *IFT172*, *KRTDAP*, *MICAL2*, *NF2*, *NRDC*, *PHACTR1*, *POPDC3*, *ROBO1*, *SPRR2D*, *TIAM2*, *TNNI3*). This is noteworthy because several studies have shown that sperm delivers to the oocyte a complex population of RNAs, which influences early embryo development and the long-term offspring phenotype [51]. These paternal RNAs regulate events both before the synthesis of embryonic gene products and beyond the zygotic genome activation [52]. Intriguingly, eleven decreased transcripts in the cryopreserved spermatozoa were localized in the centrosome, whose intact structure is essential for successful fertilization and which serves as a template for all centrioles during subsequent cell divisions, embryo development, and divisions of most adult somatic cells [53]. Compromised sperm centrosome functions are associated with decreased fertility and/or male factor infertility [54].

Furthermore, the reproductive potential of frozen sperm can also be reduced at the embryo implantation level through up-regulation of the immune-activating pathway. Mature spermatozoa express HLA class I and, above all, HLA class II, and it seems that HLA expression is an activating mechanism for the immune system of the female reproductive tract [55]. This immune response that occurs after coitus and insemination results in immunological homeostasis suitable for tolerating semen and the semi-allogeneic fetus and subsequently a successful pregnancy [56]. The level of HLA class II expression has been shown to be higher in infertile men [57] and aberrant expression has been associated with pregnancy complications such as recurrent miscarriage and pre-eclampsia [58,59]. Therefore, the higher amounts of *HLA* class II transcripts observed after cryopreservation may partially reduce the implantation success of embryos from frozen semen.

Intriguingly, along with the reduction of transcripts important for fertilization and early embryonic development, we found some transcripts changes that are suggestive of compensatory mechanisms for sperm cryoinjury, similarly to what reported by Wang et al. in blue catfish sperm [60]. In particular, the increased transcripts in cryopreserved sperm were mainly enriched in pathways involved in antigen processing and presentation. Freezing may stimulate the positive regulation of immune response. Notably, the inflammatory-related genes play a pivotal role in spermatozoa physiology and could facilitate the interaction of spermatozoa with the mucosal immune cells of the female reproductive tract [61].

Overall, the above molecular framework may give reason for the clinical evidence that is reassuring with regard to the use of cryopreserved spermatozoa in ART. As for the follow-up of offspring with cryopreserved sperm, case reports described live births from ICSI with semen stored for 21 and 40 years [62,63], and from intrauterine insemination with semen stored for 21 and 28 years [64]. Systematic analyses are also quite reassuring in that they indicate sperm cryopreservation does not adversely affect clinical, obstetric, and neonatal outcomes [65,66,67,68,69,70,71,72,73,74]. In order to fully assess the safety of sperm cryopreservation, it would be advisable to carry out broader multicentric studies with a long-term follow-up of the offspring.

We are aware that this study has some limitations due mainly to the lack of data validation in a wider sample cohort and by quantitative PCR and/or at protein level, although we have applied strict criteria for gene selection (FDR = 0). In a future study, it would be interesting to integrate the transcriptome data with the examination of sperm characteristics of warmed cryopreserved aliquots, although the results of gene ontology of transcripts significantly modified with cryopreservation are consistent with the alterations (in terms of motility, acrosome integrity, DNA fragmentation) commonly found in cryopreserved sperm. Moreover, we did not eliminate potential other cellular components besides spermatozoa from our samples, although the percentage of round cells was very low and both fresh and cryopreserved samples have this potential bias. In the future, it would be interesting to confirm these results after isolation of spermatozoa and to test whether the length of cryostorage can have any effect on the transcriptome of sperm.

The sperm cryopreservation procedure needs to be improved in the future. Possible strategies to prevent gamete damage and new approaches to sperm cryopreservation include vitrification [75]; pre-cryopreservation selection of motile spermatozoa [76]; addition of antioxidant to cryoprotectants with the aim of mitigating the possible toxic effects of reactive oxygen species generated during the freezing/thawing [77]; addition of growth factors [78]; lyophilization of sperm which would avoid the use of liquid nitrogen, allowing easy storage, packaging, and transfer of the samples [79]; and use of alternative devices for low sperm number [80]. Although vitrification seems to be the most promising approach, since it is superior to conventional freezing methods in sperm preservation with regard to motility [75], there is currently no validation of these innovations in clinical settings and we are far from defining the optimal mixture of cryoprotectants and the best freezing procedure. Further research is still needed to improve all the critical points. Moreover, it would be important to carry out future studies aimed at highlighting variations on the transcriptome in sperm by different cryoprotectants and cryopreservation protocols (i.e., vitrification and slow freezing).

Our results, and those of other omics studies, are not directly applicable to infertility treatments. Rather, studies focusing on the characterization of transcripts whose levels are changed after cryopreservation could identify potential markers of sperm susceptibility to cryoinjury. Hopefully, such markers could be used to identify samples at risk of higher cryodamage, allowing better patient counseling, and may be helpful in optimizing novel, less harmful freezing strategies.

## 4. Materials and Methods

### 4.1. Study Design, Size, Duration

Semen samples were collected from 13 normospermic men from April to May 2022. They did not have a male infertility factor and came to our center because of a female infertility factor. Eight donors (60%) had a healthy baby. The remaining 5 are still performing ART cycles.

The men enrolled in this study had a median age of 35.0 years (range: 29.0–46.0). Age and semen parameters of the 13 donors enrolled in the study are listed in Table S5.

Each sample was divided into two aliquots. One aliquot was washed in 1 mL of Gamete buffer (Cook Medical, Sydney, Australia) prewarmed at 37 °C. After centrifugation at 1400 rpm for 10 min, the pellet was transferred into the QIAzol^®^ Lysis Reagent (Qiagen, Hilden, Germany) and total RNA was immediately extracted as described below. The second aliquot was frozen and total RNA was extracted after a week of storage in liquid nitrogen. The 13 paired RNA samples passed quality control and were randomized in 4 pools, each of 6 donors, in order to minimize any man-specific variability in gene expression.

### 4.2. Sperm Cryopreservation and Thawing

After collection by masturbation, sperm samples were liquefied at room temperature for 30–60 min. Semen volume, sperm concentration and motility were evaluated following the WHO guidelines [1]. The SpermFreeze™ medium (FertiPro NV, Beernem, Belgium) was added to the sperm in drops while gently swirling (0.7 mL of medium per ml of sperm). During the 10 min incubation for equilibration at room temperature, the mixture was sucked into the CBS High Security sperm straws (Cryo Bio System, L’Aigle, France) that were placed in liquid nitrogen vapor phase for 15 min. Then the straws were quickly transferred to liquid nitrogen and stored at −196 °C for one week.

To thaw, the straws were removed from the liquid nitrogen and warmed at room temperature until the sample thawed. Then the end of the straw was cut off and the semen–medium mixture was put into a tube containing 1 mL of Gamete buffer (Cook Medical) prewarmed at 37 °C. After centrifugation at 1400 rpm for 10 min the pellet was transferred into the QIAzol^®^ Lysis Reagent (Qiagen) and processed as described below.

We checked the percentage of round cells in our samples, and it was very low, with an average 5% (range 0–20%) of the sperm count.

### 4.3. RNA Isolation and Quantification

Total RNA was extracted using the miRNeasy Micro kit (Qiagen), according to the manufacturer’s procedure. High Sensitivity RNA kit on 2200 Tape Station system (Agilent Technologies, Santa Clara, CA, USA) was used to quantify and control the quality of RNAs. The RNA samples which passed quality control check were randomized in pools.

### 4.4. Gene Expression Profiling

Five ng of total RNA purified from each pool were amplified using Ovation Pico WTA System V2 (NuGEN Technologies, San Carlos, CA, USA) and labeled by Enzymatic Labeling Kit (Agilent Technologies). Three µg of purified-Cye3-labeled cDNA were hybridized to Human GE 4x44K v2 microarrays (Agilent Technologies) at 65 °C for 17 h. Slides were washed and scanned by Agilent G2505C scanner. Raw data were extracted using Feature Extraction (FE) software v10.7, GE1_1100_Jul11 protocol (Agilent Technologies).

### 4.5. Statistical Analysis of Microarray Data

Tab-delimited text files containing FE results were acquired. The paired Significance Analysis of Microarrays (SAM) [81] was performed in R/BioConductor using the limma R package to normalize data, performing background correction and quantile normalization between arrays [82]. Array probe annotation was performed with hgug4112a.db and samr R packages [83]. Differentially expressed genes were identified by applying variance and intensity filters. Significant genes (probes with False Discovery Rate (FDR) = 0) were clustered by hierarchical clustering with average linkage and Euclidean distance measure. Probes without annotations and low counts, or those without annotations, have been removed.

### 4.6. Functional Annotation and Gene Ontology Analysis

Functional enrichment annotation analysis of the Gene Ontology (GO) categories was performed using the Database for Annotation, Visualization, and Integrated Discovery (DAVID) Bioinformatics Resources (http://david.abcc.ncifcrf.gov accessed on 4 September 2023 [84,85]. Molecular Function (MF), Biological Process (BP), and cellular components (CC) pathway analyses were performed to identify pathways significantly (*p* ≤ 0.05) over-represented in our datasets. Graphical representation (donut plots) of GO results were obtained with Ggplot2 package of R software (v. 4.2.1).

## 5. Conclusions

This paper for the first time provides a molecular explanation of the clinical evidence of reassuring ART outcomes with cryopreserved semen. These findings are noteworthy for safety issues of sperm banking, i.e., fertility preservation in men at risk of losing fertility due to gonadotoxic therapies or surgery, and sperm donation programs. 

## Data Availability

Microarray raw data have been deposited in the National Center for Biotechnology Information Gene Expression Omnibus (GEO, http://www.ncbi.nlm.nih.gov/geo/) accessed on 4 September 2023 and are accessible through GEO access number GSE225320.

## Supplementary Materials

The following supporting information can be downloaded at: https://www.mdpi.com/article/10.3390/ijms25074131/s1.
