# Supplementary material for: A Pilot Analysis of Whole Transcriptome of Human Cryopreserved Sperm
Source: Int J Mol Sci. 2024 Apr 8;25(7):4131. doi: 10.3390/ijms25074131 (PMC11012871; doi:10.3390/ijms25074131)
Supplement: Supplementary file 1 [file ijms-25-04131-s001.zip › Table S1 rev.pdf]

Table S1. List of transcripts less abundant in cryopreserved sperm in comparison with non-cryopreserved sperm.

ABCC3	ACTL7A	ACTRT1	ACTRT2	AGT
AKAP14	AKAP4	AKIRIN1	ALYREF	AMY1C
ANKEF1	ANO1	ANO2	ASGR1	ATL3
BAG1	BANF2	BPI	BRIP1	C11orf71
C12orf42	C12orf54	C16orf78	C1orf100	C1QTNF3
C2orf73	C2orf88	C9orf24	CABS1	CAMP
CAP1	CAPZA3	CAPZB	CAST	CBY2
CCDC180	CCDC185	CCDC54	CCIN	CCL15
CDRT4	CDV3	CELA3B	CEP170	CEP295NL
CHCHD3	CLMN	CLPB	CMSS1	CNN1
CPNE9	CST13P	CST8	CXorf65	CYP2R1
DCAF6	DCC	DHRS3	DNAJB8	DNAL4
DUSP21	DYNLL2	DYRK4	EEF1G	EEF2KMT
EGFLAM	ENTR1	EPB41L2	ERCC6	EXOC5
FAM161B	FAM182B	FAM20A	FAM71A	FAM71B
FAM71C	FAM71D	FAM71E1	FAM81B	FBXO39
FGL1	FLJ40194	FREM1	GLRX2	GPI
GSG1	GTPBP2	H1-7	HEMGN	HENMT1
HERPUD2	HMGB4	HMOX2	HRK	HSPA1L
IFT172	IGSF21	IQCF1	IQCF2	IQCG
ITPR3	IZUMO2	KCNIP2	KCNV2	ITPR3
KIF2B	KIF5A	KIZ	KLHL11	KLHL7
KRTDAP	LEMD1	LGALS13	LIMK2	LINC01667

LOC401176	LSM2	LYPLA1	LYRM1	LYST
MAP3K19	MAPRE3	MARCHF8	MEA1	MICAL2
MLF1	MPP3	MS4A5	NCOR1	NCOR1P1
NELFE	NF2	NRDC	NUP210P1	OAZ3
ODF2	PCP2	PDCL2	PGRMC2	PHACTR1
PI3	PKD2L1	PLCZ1	POLR3C	POPDC3
PRKAR2A	PROK2	PRSS37	PRSS58	PSMA6
RAB27B	RFPL3S	RIBC1	RNF133	RO60
ROBO1	RORA	RUBCNL	SAMD4A	SAXO1
SCP2D1	SELENOK	SERGEF	SERP2	SERPINB4
SGCA	SIRPD	SLC5A1	SMYD3	SOX9-AS1
SPATA19	SPATA32	SPATA6	SPATA6L	SPEM1
SPIN1	SPIN2B	SPPL2C	SPRR2C	SPRR2D
SPTLC1	ST6GALNAC2	STX8	SYAP1	SYN3
TBC1D21	TCF4	TEX35	TEX36-AS1	TBC1D21
TEX55	TIAM2	TIMD4	TLE4	TMCO5A
TNC	TNNI3	TRIM42	TRIP12	TSPAN16
TTC39A	TTLL10	TTLL2	TUBA4A	TUBA8
TUBGCP3	TXNDC2	UBE2DNL	UBL3	UBQLN3
UCK2	USE1	WDR20	WDR66	WFDC11
WFDC9	ZDHHC20	ZNF295-AS1	ZNRF3	
