# Supplementary material for: A Pilot Analysis of Whole Transcriptome of Human Cryopreserved Sperm
Source: Int J Mol Sci. 2024 Apr 8;25(7):4131. doi: 10.3390/ijms25074131 (PMC11012871; doi:10.3390/ijms25074131)
Supplement: Supplementary file 1 [file ijms-25-04131-s001.zip › Table S2 rev.pdf]

Table S2. List of transcripts more abundant in cryopreserved sperm in comparison with non-cryopreserved sperm.

ALKBH5	ALOX15B	AMD1	ARHGEF15	BBS5
CD74	CXCL2	CYP2B6	FOSB	HLA-DMA
HLA-DQB1	HLA-DRA	HLA-DRB1	ILDR1	MAMLD1
MYO1C	NEFH	PLAAT5	RASD1	RGS1
RPL10	RPS17	S100A4	SNAI1	TCF15
THBS1	TLE1	ZG16B		
