# Supplementary material for: A Pilot Analysis of Whole Transcriptome of Human Cryopreserved Sperm
Source: Int J Mol Sci. 2024 Apr 8;25(7):4131. doi: 10.3390/ijms25074131 (PMC11012871; doi:10.3390/ijms25074131)
Supplement: Supplementary file 1 [file ijms-25-04131-s001.zip › Table S3 rev.pdf]

Table S3. The most significantly (P<0.05) enriched Gene Ontology Biological Process (BP), cellular components (CC) and molecular function (MF) terms in transcripts decreased in cryopreserved sperm respect to non-cryopreserved sperm.

Category	Term	P-Value	Genes
GOTERM_BP_DIRECT	GO:0007283~spermatogenesis	2,40E-05	HERPUD2, SPATA6L, ODF2, LIMK2, C9ORF24, MEA1, CABS1, SPATA6, OAZ3, SPATA32, TBC1D21, PROK2, TXNDC2, CCIN, SPATA19
GOTERM_BP_DIRECT	GO:0030154~cell differentiation	1,30E-02	HEMGN, KRTDAP, CPNE9, ODF2, SYAP1, C9ORF24, GLRX2, MEA1, SPATA6, TXNDC2, TCF4, CCIN, SPATA19
GOTERM_BP_DIRECT	GO:0030036~actin cytoskeleton organization	4,60E-03	CAP1, CAPZB, CAPZA3, LIMK2, CCIN, PHACTR1, NF2
GOTERM_BP_DIRECT	GO:0000209~protein polyubiquitination	1,20E-03	PSMA6, MARCHF8, TRIP12, UBE2DNL
GOTERM_BP_DIRECT	GO:0007017~microtubule-based process	2,40E-03	DYNLL2, DNAL4, TUBA4A, TUBA8
GOTERM_BP_DIRECT	GO:0006511~ubiquitin-dependent protein catabolic process	2,90E-03	PSMA6, TRIP12, RNF133, UBQLN3
GOTERM_BP_DIRECT	GO:0007339~binding of sperm to zona pellucida	4,50E-03	HSPA1L, PRKAR2A, PRSS37

GOTERM_BP_DIRECT	GO:0007338~single fertilization	2,30E-02	ACTL7A, TUBGCP3, AKAP4
GOTERM_BP_DIRECT	GO:0030317~flagellated sperm motility	4,60E-02	TBC1D21, SPEM1, AKAP4, CABS1
GOTERM_BP_DIRECT	GO:0007286~spermatid development	4,70E-02	BRIP1, ODF2, IQCG, TUBA8
GOTERM_BP_DIRECT	GO:0008544~epidermis development	4,50E-03	KRTDAP, IFT172, SPRR2D
GOTERM_BP_DIRECT	GO:0044782~cilium organization	1,80E-02	ODF2, FAM161B, IQCG
GOTERM_BP_DIRECT	GO:0007507~heart development	2,60E-02	POPDC3, MICAL2, TNNI3
GOTERM_BP_DIRECT	GO:0050772~positive regulation of axonogenesis	2,80E-02	TIAM2, NRDC, ROBO1
GOTERM_BP_DIRECT	GO:0031032~actomyosin structure organization	3,10E-02	CNN1, EPB41L2, PHACTR1
GOTERM_BP_DIRECT	GO:0006281~DNA repair	3,60E-02	BRIP1, TRIP12, ERCC6
GOTERM_BP_DIRECT	GO:0051225~spindle assembly	5,20E-02	NCOR1, TUBGCP3, MAPRE3
GOTERM_BP_DIRECT	GO:0051085~chaperone mediated protein folding requiring cofactor	3,50E-02	HSPA1L, BAG1
GOTERM_BP_DIRECT	GO:0000077~DNA damage checkpoint	3,70E-02	BRIP1, ERCC6
GOTERM_BP_DIRECT	GO:0001707~mesoderm formation	3,90E-02	GPI, NF2
GOTERM_BP_DIRECT	GO:0006816~calcium ion transport	4,90E-02	PLCZ1, SELENOK

GOTERM_BP_DIRECT	GO:0030968~endoplasmic reticulum unfolded protein response	5,10E-02	HERPUD2, SERP2
GOTERM_CC_DIRECT	GO:0097224~sperm connecting piece	1,10E-04	SPATA6L, CAPZB, AKAP4, SPATA6
GOTERM_CC_DIRECT	GO:0005856~cytoskeleton	7,60E-04	DYRK4, ACTL7A, AKAP4, DYNLL2, TUBA4A, CNN1, CAPZB, SGCA, TBC1D21, EPB41L2, NF2, ACTRT2, SAXO1, ACTRT1
GOTERM_CC_DIRECT	GO:0005743~mitochondrial inner membrane	1,60E-03	DUSP21, CHCHD3, CABS1, SPATA19
GOTERM_CC_DIRECT	GO:0001669~acrosomal vesicle	4,50E-03	TBC1D21, ACTL7A, PRSS37, IQCF1, CABS1, TUBA8
GOTERM_CC_DIRECT	GO:0005929~cilium	6,90E-03	CEP295NL, ODF2, TTLL2, IFT172, FAM161B, DYNLL2, DNAL4, MLF1
GOTERM_CC_DIRECT	GO:0015630~microtubule cytoskeleton	7,90E-03	TEX35, MAPRE3, KIF2B, FAM161B, LYST, TUBA4A, TUBA8
GOTERM_CC_DIRECT	GO:0005874~microtubule	9,50E-03	ODF2, KIF5A, CEP170, TUBGCP3, KIF2B, DYNLL2, DNAL4, TUBA4A, TUBA8
GOTERM_CC_DIRECT	GO:0097225~sperm midpiece	1,00E-02	TBC1D21, IFT172, AKAP4, SPATA19

GOTERM_CC_DIRECT	GO:0048471~perinuclear region of cytoplasm	1,60E-02	ZDHHC20, STX8, LIMK2, SYAP1, C9ORF24, SLC5A1, AKAP4, MLF1, PLCZ1, SPATA32, KLHL7, KIF5A, MAPRE3, NF2
GOTERM_CC_DIRECT	GO:0005737~cytoplasm	2,00E-02	DYRK4, CLPB, SYAP1, MEA1, DCAF6, ROBO1, RO60, HENMT1, SGCA, CYP2R1, KIF5A, PHACTR1, BANF2, CAST, CAP1, CDV3, SERPINB4, LYPLA1, MLF1, TUBA4A, IQCG, EEF1G, PSMA6, ANO1, DUSP21, LGALS13, MAPRE3, BPI, EXOC5, KIF2B, FAM20A, SPEM1, ACTRT2, KIZ, ACTRT1, ACTL7A, ITPR3, CST8, AKAP4, OAZ3, BRIP1, CLMN, PRKAR2A, BAG1, EEF2KMT, MARCHF8, DNAJB8, TXNDC2, RNF133, SPRR2D, HSPA1L, ODF2, KCNIP2, LIMK2, ALYREF, PRSS37, MICAL2, LSM2, UCK2, PDCL2, TUBGCP3, NF2, SERGEF, DNAL4, TUBA8
GOTERM_CC_DIRECT	GO:0031514~motile cilium	2,20E-02	ACTL7A, AKAP4, SAXO1, CABS1, IQCG
GOTERM_CC_DIRECT	GO:0042995~cell projection	2,50E-02	ANO1, EGFLAM, ODF2, NF2, KIZ, CAMP
GOTERM_CC_DIRECT	GO:0036126~sperm flagellum	2,80E-02	ODF2, SAXO1, SPATA19, IQCG

GOTERM_CC_DIRECT	GO:0005813~centrosome	3,10E-02	CEP295NL, ODF2, LIMK2, PRKAR2A, CEP170, TUBGCP3, KIF2B, ENTR1, KIZ, DYNLL2, TTC39A
GOTERM_CC_DIRECT	GO:0097228~sperm principal piece	3,60E-02	IFT172, AKAP4, CABS1
GOTERM_MF_DIRECT	GO:0005509~calcium ion binding	2,90E-06	PLCZ1, KCNIP2, AMY1C, PKD2L1, ITPR3, ANKEF1, CABS1
GOTERM_MF_DIRECT	GO:0003779~actin binding	3,90E-04	CAP1, CNN1, CAPZB, CAPZA3, SPATA32, TBC1D21, CLMN, EPB41L2, MICAL2, TNNI3, PHACTR1, NF2
GOTERM_MF_DIRECT	GO:0005515~protein binding	3,60E-03	CLPB, TTLL10, SPPL2C, C2ORF88, SYAP1, TNC, PKD2L1, RORA, DCAF6, CHCHD3, HENMT1, KIF5A, PROK2, FAM81B, CAST, TLE4, STX8, TEX55, GTPBP2, IQCF2, LEMD1, TUBA4A, PCP2, TBC1D21, LGALS13, RUBCNL, SPIN2B, FAM161B, FREM1, ACTL7A, KLHL11, ITPR3, SLC5A1, OAZ3, CNN1, BRIP1, KCNV2, PRKAR2A, EPB41L2, BAG1, TXNDC2, SMYD3, HMOX2, AKIRIN1, ODF2, ALYREF, NRDC, FBXO39, MICAL2, RAB27B, ANKEF1, LSM2, H1-7, TCF4, TUBGCP3, NF2, SERGEF, DNAL4, TUBA8, HRK, HERPUD2, GPI, DYRK4, WFDC9,

			CCDC121, CBY2, MEA1, USE1, LYST, HMGB4, ASGR1, ROBO1, SERP2, C1QTNF3, UBL3, SPTLC1, SGCA, CAPZB, AKAP14, GSG1, SPIN1, CCIN, CEP170, PHACTR1, NELFE, CAP1, TMCO5A, LYPLA1, DCC, C9ORF24, GLRX2, DYNLL2, ANO2, MLF1, EEF1G, PSMA6, ANO1, NCOR1, DUSP21, MAPRE3, EXOC5, FAM20A, SAXO1, KIZ, HEMGN, ATL3, FGL1, WDR20, SELENOK, AKAP4, ZNRF3, PGRMC2, EGFLAM, MARCHF8, EEF2KMT, TNNI3, ENTR1, CCDC185, TRIM42, RIBC1, UBQLN3, CDRT4, MPP3, HSPA1L, KCNIP2, LIMK2, SAMD4A, AGT, CMSS1, SPATA32, POLR3C, KLHL7, TRIP12, ERCC6
GOTERM_MF_DIRECT	GO:0005227~calcium activated cation channel activity	9,50E-03	ANO1, PKD2L1
GOTERM_MF_DIRECT	GO:0005229~intracellular calcium activated chloride channel activity	1,10E-02	ANO1, ANO2

GOTERM_MF_DIRECT	GO:0004867~serine-type endopeptidase inhibitor activity	1,90E-02	WFDC11, WFDC9, SERPINB4, PI3, AGT
GOTERM_MF_DIRECT	GO:0008574~ATP-dependent microtubule motor activity, plus-end-directed	2,60E-02	KIF5A, DYNLL2, DNAL4
GOTERM_MF_DIRECT	GO:0031625~ubiquitin protein ligase binding	4,10E-02	HSPA1L, STX8, BAG1
GOTERM_MF_DIRECT	GO:0005254~chloride channel activity	4,20E-02	ANO1, ANO2
GOTERM_MF_DIRECT	GO:0005262~calcium channel activity	4,40E-02	PKD2L1, ITPR3
GOTERM_MF_DIRECT	GO:0061630~ubiquitin protein ligase activity	4,90E-02	MARCHF8, TRIP12, RNF133
GOTERM_MF_DIRECT	GO:0044183~protein binding involved in protein folding	5,20E-02	HSPA1L, DNAJB8
