# Supplementary material for: A Pilot Analysis of Whole Transcriptome of Human Cryopreserved Sperm
Source: Int J Mol Sci. 2024 Apr 8;25(7):4131. doi: 10.3390/ijms25074131 (PMC11012871; doi:10.3390/ijms25074131)
Supplement: Supplementary file 1 [file ijms-25-04131-s001.zip › Table S4 rev.pdf]

Table S4. The most significantly (P<0.05) enriched Gene Ontology Biological Process (BP), cellular components (CC) and molecular function (MF) terms in transcripts increased in cryopreserved sperm respect to non-cryopreserved sperm.

Category	Term	PValue	Genes
GOTERM_BP_DIRECT	GO:0019886~antigen processing and presentation of exogenous peptide antigen via MHC class II	1,0E-7	CD74, HLA-DMA, HLA-DRA, HLA-DRB1, HLA-DQB1
GOTERM_BP_DIRECT	GO:0019882~antigen processing and presentation	4,2E-7	CD74, HLA-DMA, HLA-DRA, HLA-DRB1, HLA-DQB1
GOTERM_BP_DIRECT	GO:0002503~peptide antigen assembly with MHC class II protein complex	1,6E-6	HLA-DMA, HLA-DRA, HLA-DRB1, HLA-DQB1
GOTERM_BP_DIRECT	GO:0002504~antigen processing and presentation of peptide or polysaccharide antigen via MHC class II	2,7E-6	HLA-DMA, HLA-DRA, HLA-DRB1, HLA-DQB1
GOTERM_BP_DIRECT	GO:0050870~positive regulation of T cell activation	1,4E-5	HLA-DMA, HLA-DRA, HLA-DRB1, HLA-DQB1
GOTERM_BP_DIRECT	GO:0050778~positive regulation of immune response	1,8E-5	HLA-DMA, HLA-DRA, HLA-DRB1, HLA-DQB1

GOTERM_BP_DIRECT	GO:0006955~immune response	5,8E-5	CD74, HLA-DMA, RGS1, HLA-DRA, THBS1, HLA-DRB1, HLA-DQB1
GOTERM_BP_DIRECT	GO:0002250~adaptive immune response	2,5E-2	HLA-DMA, HLA-DRA, HLA-DRB1, HLA-DQB1
GOTERM_CC_DIRECT	GO:0042613~MHC class II protein complex	3,4E-8	CD74, HLA-DMA, HLA-DRA, HLA-DRB1, HLA-DQB1
GOTERM_CC_DIRECT	GO:0071556~integral component of luminal side of endoplasmic reticulum membrane	7,1E-6	CD74, HLA-DRA, HLA-DRB1, HLA-DQB1
GOTERM_CC_DIRECT	GO:0098553~lumenal side of endoplasmic reticulum membrane	7,1E-6	CD74, HLA-DRA, HLA-DRB1, HLA-DQB1
GOTERM_CC_DIRECT	GO:0030658~transport vesicle membrane	2,4E-5	CD74, HLA-DRA, HLA-DRB1, HLA-DQB1
GOTERM_CC_DIRECT	GO:0012507~ER to Golgi transport vesicle membrane	5,6E-5	CD74, HLA-DRA, HLA-DRB1, HLA-DQB1
GOTERM_CC_DIRECT	GO:0030669~clathrin-coated endocytic vesicle membrane	1,0E-4	CD74, HLA-DRA, HLA-DRB1, HLA-DQB1
GOTERM_CC_DIRECT	GO:0030666~endocytic vesicle membrane	1,2E-4	CD74, HLA-DRA, HLA-DRB1, HLA-DQB1
GOTERM_CC_DIRECT	GO:0032588~trans-Golgi network membrane	3,6E-4	CD74, HLA-DRA, HLA-DRB1, HLA-DQB1
GOTERM_CC_DIRECT	GO:0031902~late endosome membrane	1,0E-3	HLA-DMA, HLA-DRA, HLA-DRB1, HLA-DQB1

GOTERM_CC_DIRECT	GO:0009986~cell surface	1,4E-3	CD74, HLA-DMA, HLA-DRA, THBS1, HLA-DRB1, HLA-DQB1
GOTERM_CC_DIRECT	GO:0005765~lysosomal membrane	1,8E-3	CD74, HLA-DMA, HLA-DRA, HLA-DRB1, HLA-DQB1
GOTERM_CC_DIRECT	GO:0010008~endosome membrane	5,3E-3	HLA-DMA, HLA-DRA, HLA-DRB1, HLA-DQB1
GOTERM_CC_DIRECT	GO:0070062~extracellular exosome	2,2E-2	CD74, HLA-DMA, HLA-DRA, HLA-DRB1, HLA-DQB1
GOTERM_MF_DIRECT	GO:0023026~MHC class II protein complex binding	5,6E-8	CD74, HLA-DMA, HLA-DRA, HLA-DRB1, HLA-DQB1
GOTERM_MF_DIRECT	GO:0032395~MHC class II receptor activity	6,5E-7	HLA-DMA, HLA-DRA, HLA-DRB1, HLA-DQB1
GOTERM_MF_DIRECT	GO:0042605~peptide antigen binding	3,8E-5	HLA-DMA, HLA-DRA, HLA-DRB1, HLA-DQB1
