# Supplementary material for: A Pilot Analysis of Whole Transcriptome of Human Cryopreserved Sperm
Source: Int J Mol Sci. 2024 Apr 8;25(7):4131. doi: 10.3390/ijms25074131 (PMC11012871; doi:10.3390/ijms25074131)
Supplement: Supplementary file 1 [file ijms-25-04131-s001.zip › Table S5.pdf]

Table S5. Age and semen parameters of the 13 donors enrolled in the study.

ID	Age (years)	Volume (ml)	Sperm concentration (10^6 per ml)	Progressive Motility (%)	Total motility (%)	Round cells (%)
1F	44	4.5	72	60	70	20
2F	34	4.0	69	55	60	4
4F	29	2.5	116	70	75	5
9F	33	3.4	22	35	40	10
10F	46	1.5	27	40	60	0
11F	35	5.0	126	55	65	4
12F	42	3.5	137	60	70	4
13F	39	2.0	83	50	60	1
16F	33	3.0	16	60	60	0
17F	45	4.0	71	55	55	0
18F	37	2.5	47	57	72	0
19F	34	1.5	23	55	55	2
20F	34	3.0	19	55	65	20
